# South Asia’s diabetes crisis needs families: how can we advance from informal care to integrated engagement?

**DOI:** 10.1016/j.lansea.2025.100607

**Published:** 2025-05-26

**Authors:** Shahmir H. Ali, Sudip Bhattacharya, Abhijit Chanda, Biswadeep Dhar

**Affiliations:** a Saw Swee Hock School of Public Health, National University of Singapore, Singapore; b Department of Community and Family Medicine, All India Institute of Medical Sciences Deoghar (AIIMS Deoghar), Deoghar, Jharkhand, India; c Department of Endocrinology and Diabetes, Medical Superspecialty Hospital, Kolkata, West Bengal, India; d Department of Human Ecology, University of Maryland Eastern Shore, Princess Anne, MD, USA

**Keywords:** South Asia, Diabetes, Chronic disease care, Family, Social support, Health system, Digital health

## Abstract

Type 2 diabetes (T2D) is an escalating concern in South Asia, with prevalence surging over the past two decades. Family members often significantly influence T2D outcomes and management, yet involvement remains informal, unstructured, and unrecognized within healthcare systems. This viewpoint calls for a more structured, equitable approach to family engagement in T2D care, outlining three models (family-supported, family-wide, and family-led) that can optimize the role of family in T2D care. Given the diversity of household structures, interventions must be adaptable to varying family dynamics. While family involvement can enhance care, challenges such as cultural barriers, gender and age disparities, and inconsistent healthcare guidance must be addressed. Providers need training and clear protocols to engage families, while policies must ensure that caregivers are equipped with adequate support. Digital tools, including social media and telemedicine, offer promising ways to enhance family support in T2D management. Ultimately, South Asia must move beyond reliance on informal care to system-integrated family engagement that recognizes and empowers those often at the frontlines of care.

## Introduction

South Asia is facing a rapidly escalating diabetes crisis, with prevalence rates more than doubling over the past two decades.^1^ Today, 22.3% of adults in India, Pakistan, Bangladesh, and Sri Lanka have diabetes, with urban areas experiencing even higher rates, exceeding 30% in some major cities.^1^ Nearly half of all cases remain undiagnosed, increasing the risk of cardiometabolic complications leading to cardiovascular disease, neuropathy, kidney failure, and cancer.^2^^,^^3^ Much of this rise is driven by the increasing prevalence of type 2 diabetes (T2D),^1^ which is characterized by insulin resistance and progressive insulin deficiency, primarily driven by lifestyle factors like poor diet, physical inactivity, and obesity. While T2D does not always require insulin, its management often involves multiple daily care activities, including maintaining a healthy diet, engaging in regular physical activity, ensuring medication adherence, and monitoring for long-term complications like cardiovascular disease, kidney issues, and neuropathy.^4^ Given strained healthcare systems, T2D care often relies not only on formal providers but also on families, who play a crucial role in self-management—especially in low-resource settings where access to care is limited.^5^^,^^6^

Across South Asia, families take an active, hands-on role in nearly every aspect of T2D care, from medication adherence and meal planning to physical activity encouragement and financial support.^7^, ^8^, ^9^ Multigenerational households create shared caregiving responsibilities, with relatives reminding individuals with T2D to take medications, accompanying them to hospital visits, and monitoring dietary choices.^7^, ^8^, ^9^ Spouses, parents, and children also influence lifestyle behaviors (including diet, exercise, stress, and sleep) which are central to T2D prevention and long-term T2D management. Moreover, it’s important to recognize that in South Asia, women (wives, daughters, daughters-in-law, etc.) bear much of the daily burden of T2D care while managing household duties, yet their contributions often go unrecognized despite the physical and emotional demands.^9^ Ultimately, T2D management often becomes a shared responsibility in South Asia, with families not only supporting daily care but also shaping long-term health outcomes through their involvement and influence.

We must also acknowledge that family members can at times hinder T2D management as well. Deep-rooted dietary traditions may make dietary changes difficult, especially when meals are prepared for shared consumption, or when those responsible for cooking may lack the awareness or capacity to align meals with diabetes care. Protective caregiving practices may discourage physical activity, reinforcing sedentary behaviors that worsen outcomes.^10^^,^^11^ Evidence from Bangladesh and other settings in Asia suggests that family members frequently take on caregiving responsibilities in hospital settings without proper training, which may lead to uncertainty in administering care, reliance on informal learning, and potential risks to patient safety.^12^ These examples highlight that families are not just passive observers but active participants in shaping T2D management, for better or worse.

## Rethinking and reevaluating family engagement: exploring new models for T2D care in South Asia

**Fig. 1:**
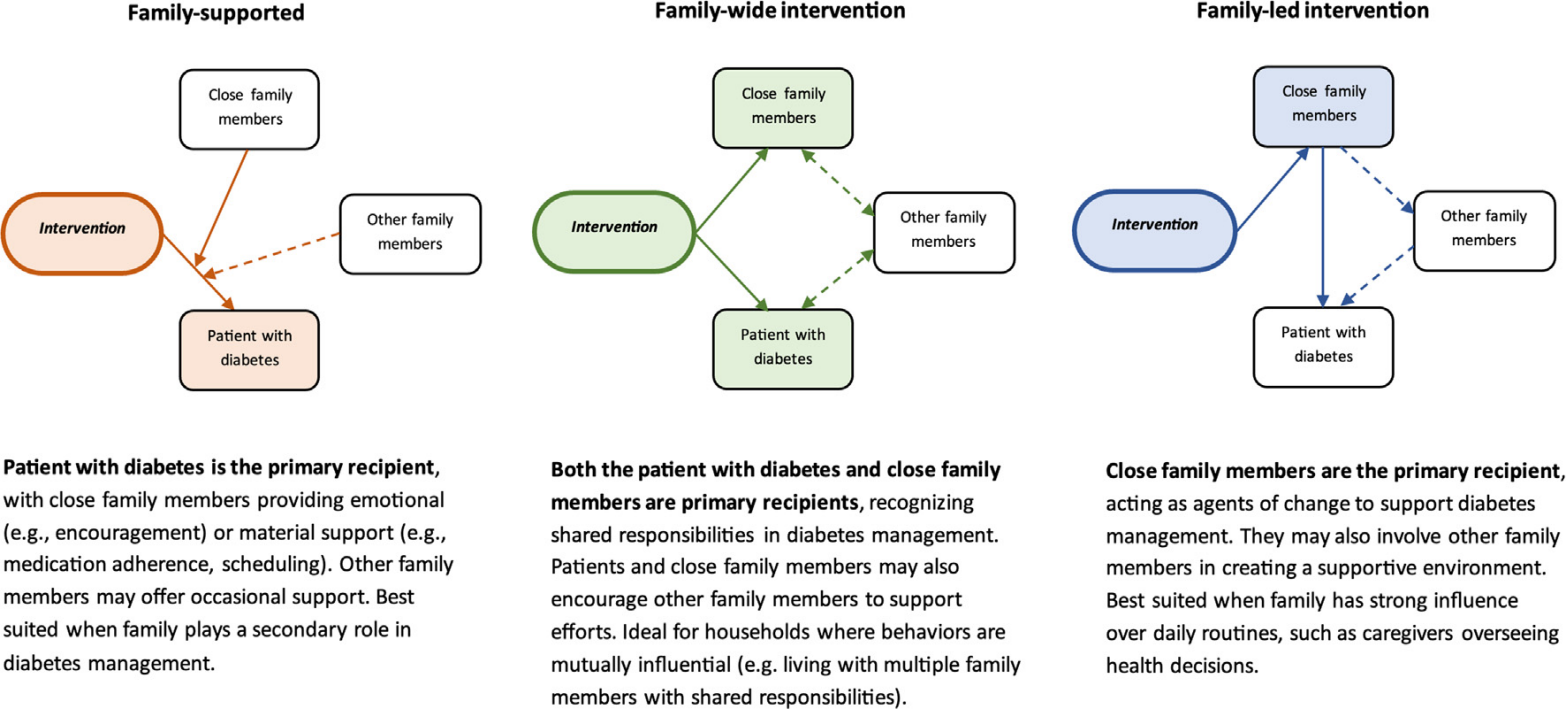
Types of family engagement models that can be considered in supporting diabetes management and prevention in South Asia.

Recognizing this profound importance of family in T2D management, the Research Society for the Study of Diabetes in India (RSSDI) has recently emphasized the need to actively involve family members in T2D care through education, counseling, and shared decision-making, ensuring they can support medication adherence, lifestyle changes, and emotional well-being.^13^ While family involvement in T2D care is often not a formalized component of interventions, several programs in South Asia have begun integrating family members in varying capacities, aligning with three emerging models of family engagement: family-supported, family-wide, and family-led approaches (Fig. 1).

The family-supported model involves family members providing emotional, logistical, or financial support while the individual living with T2D retains primary responsibility for managing their health.^7^ This approach is beneficial for individuals who manage their condition independently but could benefit from additional support with, for example, medication adherence, symptom tracking, and clinic visits. The INDEPENDENT study in India, which integrated T2D and depression care, demonstrated this model by engaging family members to encourage medication adherence, monitor symptoms, and assist with healthcare expenses while individuals with T2D received behavioral support from care coordinators.^14^ The family-wide intervention model extends beyond individual support by emphasizing shared responsibility among household members, making it particularly effective in multigenerational families where food, physical activity, and health routines are often collectively determined. The Kerala Diabetes Prevention Program (K-DPP) implemented this model by actively involving family members in structured interventions.^15^ They participated in educational sessions, joined group exercise activities, and contributed to household dietary changes, reinforcing healthier lifestyles at a family level.

While family-supported and family-wide models have been explored in T2D care in South Asia, the family-led model remains largely understudied despite its significant potential. Unlike traditional models, this approach designates a trusted relative to take charge of key aspects of care, making it especially beneficial for individuals with mobility issues, low health literacy, or reduced motivation. In South Asia, where family members play a central role in decision-making and caregiving, a structured family-led approach could be especially impactful. A randomized controlled trial in Thailand showed the potential of this approach: when family members received structured T2D education and mobile app-based support, their relatives living with T2D saw significant improvements in glycemic control, medication adherence, and self-management, despite not being the direct recipients.^16^ Yet, even with South Asia’s strong kinship networks and often multigenerational household structures, family-led T2D interventions remain largely underexplored or understudied. Investing in this approach and adapting it to South Asian cultural norms and caregiving structures could shift family involvement from being an informal source of support to a structured, active driver of better health outcomes.

## Beyond one-size-fits-all: flexible, system-ready family engagement in T2D care

A key challenge in integrating family members into T2D care is ensuring their involvement is practical and adaptable across different intervention models. While some relatives take an active role, others may feel overwhelmed or reluctant, yet they still influence an individual’s health through diet, emotional support, and household routines.^8^ Interventions must accommodate these varying levels of engagement, ensuring that all family members can contribute meaningfully; some may be able to fully manage the care of a person living with T2D, while others, even with limited time or willingness, can still reinforce healthy behaviors or offer encouragement. Likewise, each model of family engagement brings its own strengths and limitations. Family-led interventions may provide consistent, hands-on support but risk undermining the autonomy of the person living with T2D, especially in situations of family conflict. Family-wide or family-supported models may help preserve agency but require strategies and effort to keep family members meaningfully engaged in structured, supportive roles. When it comes to concerns around navigating complex family dynamics, one promising approach is to prepare individuals living with T2D themselves to engage their families more effectively. A culturally adapted coping skills program for Chinese American immigrants with T2D exemplifies this by shifting the focus from medical education to relationship-building. Instead of training family members, the intervention taught individuals with T2D problem-solving and communication strategies to navigate caregiving tensions, overcome family resistance, and gain support for lifestyle changes.^17^ This model prioritized relationship-building, integrating T2D care through stronger family communication. In South Asia, where families can often be large and shaped by diverse interaction patterns and relationships,^8^^,^^9^ culturally relevant, adaptive strategies could ensure that even minimally engaged relatives contribute to long-term disease management.

As family involvement in T2D care expands, the healthcare system must be equipped to support this shift. Family-centered care requires healthcare professionals to engage families through culturally sensitive communication and structured collaboration. However, a study on Indian nurses highlights gaps in training, standardized protocols, and institutional support that hinder meaningful integration.^18^ Many providers face unclear expectations about family roles, limited caregiver education resources, and a lack of formal guidelines, leading to inconsistent engagement. Concerns also arise around overprotection, diminished autonomy of individuals with T2D, and misaligned family support that can undermine treatment adherence.^19^ In such cases, it is essential that family-involved interventions build upon existing family dynamics rather than contribute to conflict. Preferences and comfort of individuals living with T2D should remain central; even in family-led models, patients must continue to engage directly with other key stakeholders in their care, particularly healthcare professionals, and have opportunities to provide ongoing feedback on the nature and extent of family involvement. Interventions should remain flexible and responsive, allowing for transitions between different models of engagement or a step back from family involvement altogether if circumstances change.

Addressing these barriers requires systemic reforms that equip providers with the skills, protocols, and tools to effectively involve families in T2D care. Specialized family-engagement training programs have improved provider confidence and T2D outcomes,^20^ though evidence specific to T2D care for older adults and among South Asian communities remains limited. Beyond training, structured protocols are crucial for standardizing family involvement. Studies show that clear guidelines, such as engagement checklists or family meetings where providers, individuals with T2D, and caregivers discuss treatment goals, improve T2D self-management and reduce hospital readmissions.^21^

Finally, as integrated models of family engagement in T2D care are explored, they must be designed equitably, ensuring family members are supported rather than overburdened. Caregiving should not be treated as an assumed duty but recognized and reinforced through policies that offer financial relief, training, workplace accommodations, and community-based services. Lessons from across Asia highlight how such policies, like Korea’s Family Care Leave and China’s national eldercare standards, can reduce strain and promote more sustainable support.^22^ At the same time, caregiving responsibilities remain unevenly distributed, with women shouldering a disproportionate share.^9^ These responsibilities often intersect with age and family hierarchies, where expectations to maintain harmony and dereference to the opinions and approval of more senior or male relatives can shape behaviors and what issues are prioritized.^10^ This can create pressure to conform, even when doing so conflicts with one’s own health needs or medical advice. At the same time, broader shifts such as urbanization, out-migration, and evolving norms around filial duty are reshaping traditional caregiving patterns, with many families moving away from joint household arrangements toward more nuclear or independent living setups, and support systems becoming increasingly fluid, diverse, and dynamic.^11^ Recognizing these realities is essential to tailoring family engagement strategies that reflect local contexts, respond to gendered, age-based, and relational family norms, and at the same time make efforts to promote more equitable involvement of diverse family members across these varying contexts in balanced and sustainable engagement mechanisms.

## The digital frontier: mobilizing families through technology

As urbanization spreads across South Asia, families are becoming more geographically dispersed, challenging traditional caregiving roles. At the same time, rapid digitalization is transforming how families stay connected, with platforms like WhatsApp, video calls, and social media not only preserving family interactions but also redefining how health support is being provided. In T2D care, digital tools are already improving disease management through connected glucose meters, telemedicine, digital health records, and mobile health (mHealth) interventions.^23^ Initiatives such as India’s Ayushman Bharat Digital Mission and Bangladesh’s Digital Health Strategy highlight efforts to integrate digital tools into healthcare, creating more accessible systems.^24^^,^^25^ A recent example is Diabetes Connect, a WhatsApp-based program offering free T2D education, personalized nutrition guidance, and expert-backed medical updates.^26^ While digital health efforts in South Asia have focused on individual care, technology’s growing role in family interactions presents an untapped opportunity. Given how central digital platforms have become in daily life, they hold immense potential for developing family-involved T2D care strategies suited to South Asia’s evolving family dynamics.

Social media platforms can enhance family involvement in T2D care through group messaging, live video sessions, automated reminders, and peer-support networks. WhatsApp, Facebook groups, and digital communities facilitate real-time monitoring, knowledge-sharing, and emotional support. In Pakistan, a WhatsApp-assisted intervention paired individuals with T2D with a nominated peer (often family members), who reinforced dietary changes, physical activity, and treatment adherence after an education program.^27^ In India, WhatsApp has been used for medical knowledge-sharing among physicians, a model that could be adapted for family-based T2D support.^28^ Outside of South Asia, other case studies highlight innovative approaches. In China, a WeChat-based intervention combined structured goal-setting, real-time reminders, and interactive learning modules to enhance T2D self-care.^29^ Families monitored key health behaviors, engaged with customized educational content, and received tailored reminders, fostering ongoing participation beyond traditional information-sharing. As South Asia explores family-centered digital strategies, such innovations can transform social media from a passive knowledge source into an interactive tool for sustained family involvement in care.

As digital health interventions advance, chatbots offer a scalable solution for T2D care, particularly in resource-limited settings like South Asia, where healthcare infrastructure is strained. Chatbots have already enhanced chronic disease management by guiding individuals on diet, exercise, medication adherence, and glucose monitoring.^30^ A real-world study in India demonstrated significant improvements in clinical T2D outcomes through a 16-week mobile-based, AI-supported self-management program delivered via chatbot.^31^ The Wellthy CARE chatbot sent daily interactive messages with personalized guidance on key self-care behaviors. However, the intervention focused on engaging individuals with T2D, and the potential to leverage chatbots to involve social and family networks remains largely untapped. Future family-inclusive T2D chatbots could support household involvement by delivering structured, conversational guidance on blood sugar monitoring, diet, complications, and emotional support. Chatbot features could also enable individuals with T2D and their family members to share personalized messages, reminders, or culturally tailored tips, facilitating more coordinated, interactive, and supportive care. AI-driven advancements would allow chatbots to adapt interactions, making them more personalized and culturally relevant for South Asian households.

## Conclusion: a new vision for family-engaged T2D care

The rising T2D burden in South Asia calls for a fundamental shift in how we approach care. Families play a defining role in shaping health behaviors, yet their involvement remains largely informal and unstructured. Moving forward, T2D interventions must intentionally and equitably integrate family networks, equipping them with structured tools, education, and support systems that enable them to actively contribute to disease management. This means going beyond simply encouraging family participation in care to designing practical, adaptable models that harness their influence in sustaining long-term health improvements. Digital health innovations, family-led strategies, and culturally embedded behavioral interventions offer promising pathways to scale these efforts. At the same time, rigorous research and evaluation are essential to refining what works, for whom, and in what contexts. Indeed, we must also remember that South Asia is not a monolith; alongside differences in family structures and caregiving norms, variations in language, religion, cultural practices, economic conditions, environmental contexts, and health infrastructure must also be considered. These factors necessitate careful adaptation of family-based models through localized formative research to ensure cultural relevance and effectiveness. Ultimately, the future of T2D care in South Asia cannot rely on individuals living with the disease alone. We must embrace families as co-architects of better health outcomes, equitably empowering them as partners in care.

## Contributors

SHA initiated and conceptualized the research idea, with SB, AC, and BD helping refine the topic. SHA led the literature review and manuscript writing, while SB, AC, and BD contributed to reviewing and editing the manuscript. All authors read and approved the paper.

## Declaration of interests

The authors declare no conflict of interests.
